# Pathway analysis of kidney cancer using proteomics and metabolic profiling

**DOI:** 10.1186/1476-4598-5-64

**Published:** 2006-11-24

**Authors:** Bertrand Perroud, Jinoo Lee, Nelly Valkova, Amy Dhirapong, Pei-Yin Lin, Oliver Fiehn, Dietmar Kültz, Robert H Weiss

**Affiliations:** 1 Genome Center, University of California, Davis, CA, USA; 2 Animal Science, University of California, Davis, CA, USA; 3 Division of Nephrology, Department of Internal Medicine, University of California, Davis, CA, USA; 4 Department of Veterans' Affairs Northern California Health Care System, Mather, CA, USA

## Abstract

**Background:**

Renal cell carcinoma (RCC) is the sixth leading cause of cancer death and is responsible for 11,000 deaths per year in the US. Approximately one-third of patients present with disease which is already metastatic and for which there is currently no adequate treatment, and no biofluid screening tests exist for RCC. In this study, we have undertaken a comprehensive proteomic analysis and subsequently a pathway and network approach to identify biological processes involved in clear cell RCC (ccRCC). We have used these data to investigate urinary markers of RCC which could be applied to high-risk patients, or to those being followed for recurrence, for early diagnosis and treatment, thereby substantially reducing mortality of this disease.

**Results:**

Using 2-dimensional electrophoresis and mass spectrometric analysis, we identified 31 proteins which were differentially expressed with a high degree of significance in ccRCC as compared to adjacent non-malignant tissue, and we confirmed some of these by immunoblotting, immunohistochemistry, and comparison to published transcriptomic data. When evaluated by several pathway and biological process analysis programs, these proteins are demonstrated to be involved with a high degree of confidence (p values < 2.0 E-05) in glycolysis, propanoate metabolism, pyruvate metabolism, urea cycle and arginine/proline metabolism, as well as in the non-metabolic p53 and FAS pathways. In a pilot study using random urine samples from both ccRCC and control patients, we performed metabolic profiling and found that only sorbitol, a component of an alternative glycolysis pathway, is significantly elevated at 5.4-fold in RCC patients as compared to controls.

**Conclusion:**

Extensive pathway and network analysis allowed for the discovery of highly significant pathways from a set of clear cell RCC samples. Knowledge of activation of these processes will lead to novel assays identifying their proteomic and/or metabolomic signatures in biofluids of patient at high risk for this disease; we provide pilot data for such a urinary bioassay. Furthermore, we demonstrate how the knowledge of networks, processes, and pathways altered in kidney cancer may be used to influence the choice of optimal therapy.

## Background

While accounting for only 3% of cancer incidence and mortality in the US, kidney cancer (renal cell carcinoma; RCC) is the sixth leading cause of cancer death in the US. Early diagnosis of kidney-localized RCC is associated with a quite favorable prognosis (89%), but patients who have this disease often present with few signs, symptoms, or laboratory abnormalities and are frequently (~30%) diagnosed at the metastatic stage when the prospects for cure are dismal (9%)[1]. The incidence of RCC in the US, as well as its associated mortality rates, are increasing[2], and the mortality rate has not improved significantly, most likely because currently available therapies for metastatic disease are relatively ineffective. Thus, novel and convenient diagnostic tests for this disease which can be utilized early in its course before metastasis, such as those which utilize readily accessible biofluids, are clearly needed.

We and others have previously identified tissue markers of RCC which have prognostic value [3-5], yet there are few extant studies which define any diagnostic protein or metabolite in RCC patient biofluids[6]. Due to its intimate association with the principal biofluid, urine, kidney cancer appears exceptionally well suited for studies to identify tumor markers in this material. In this study, we have undertaken a comprehensive computational analysis of tissue proteomic data to discover pathways and networks involved in clear cell RCC (ccRCC) oncogenesis and progression. Furthermore, using metabolomic analysis, we provide evidence in the urine of alterations in those pathways which we have identified, constituting a first step towards elucidation of an urinary "metabolic signature" of ccRCC which will prove useful for kidney cancer diagnostic testing of high risk patients in the clinic. Our finding of striking homogeneity among the samples evaluated based on statistical analysis suggests the feasibility of using relatively low sample numbers in future ccRCC proteomic analyses.

## Results

### Proteomic analysis of clear cell RCC tissue

Both tumor and adjacent normal (generally cortical) tissue from the same kidney was obtained from four patients (eight samples total) who had undergone nephrectomy for renal masses and had the histological diagnosis of clear cell RCC. The distribution was two male patients with tumor grades 1 and 2, stage I and two female patients with tumor grade 2, stages II and III; age ranges were from 32 to 79 years old. Only TNM staging and tumor grade were available for all tissues. Despite these differences in grade and stage, subsequent proteomic analysis yielded remarkably similar and statistically highly significant findings, suggesting homogeneity of biochemical processes in ccRCC, as well as the veracity of our data, using a relatively small sample number.

Proteins from these tissues were extracted and purified using buffers optimized for maximum protein recovery from renal tissue, and separated by high-resolution 2-dimensional gel electrophoresis as described in Methods. Proteins identified as significantly overexpressed and underexpressed in tumors as compared to their corresponding control tissues (Delta 2D analysis, see Materials and Methods) were extracted from gels, in-gel digested with trypsin, and prepared for mass spectrometric (MS) analysis. Proteins were identified by peptide mass fingerprinting (Mascot), MS/MS *de novo *sequencing and BLASTP2 sequence matching[7].

**Figure 1:**
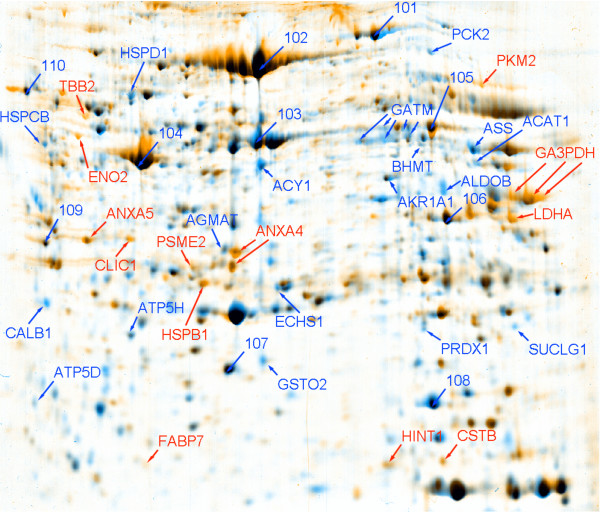
Proteomic analysis of RCC. 2-D gel electrophoresis shows proteins decreased (blue) or increased (red) in ccRCC as compared to adjacent normal renal tissue. Numbers 101 – 110 refer to the 10 internal standard spots used for normalization (see Materials and Methods)

**Figure 2:**
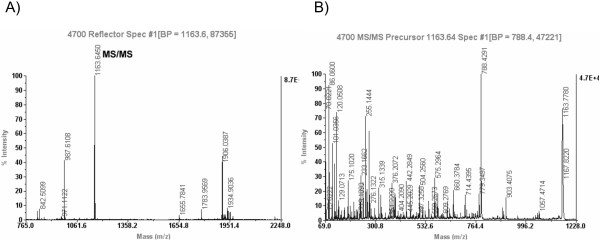
MS and MS/MS spectra for HSP27. A) MS spectrum of HSP27. The peptide whose MS/MS spectrum is shown in panel B is indicated. B) MS/MS spectrum of the peptide ion m/z 1163 obtained in CID mode.

When examined for up- or down-regulation in ccRCC as compared to adjacent control renal tissue, we identified 46 spots by MS with a high degree of confidence (Fig. 1; average Mascot score of 459 and average sequence coverage of 47%; see Supplemental Table 1 for a list of all the identified proteins). Of the 46 identified proteins (Supplemental Table 1), 31 showed significant changes with p value < 0.05 (Table 1). Quantification of up-regulated proteins in tumor tissue showed increases from 2-fold to over 30-fold when compared to expression in normal control renal tissue. Examples of MS and MS/MS spectra for one of the proteins identified, Hsp27 (official NCBI symbol HSBP1), are shown in Fig. 2 and an annotation of *de novo *amino acid sequence is shown in Supplemental Fig. 1. This protein is also labeled in Fig. 1 as HSBP1.

**Table 1:** Proteins from our proteomic analysis which were significantly different in ccRCC as compared to normal kidney tissue.

**Symbol**	**Protein Name**	**SwissProt ID**	**Entrez Gene ID**	**Ratio RCC: normal kidney**	**Minimum Paired t-test**
SUCLG1	succinate-CoA ligase, GDP-forming, alpha subunit	P53597	8802	**0.2**	0.0000
HSPCB	heat shock 90kDa protein 1, beta	P08238	3326	**0.4**	0.0001
ALDOB	aldolase B, fructose-bisphosphate	P05062	229	**0.1**	0.0001
PRDX1	peroxiredoxin 1	Q06830	5052	**0.3**	0.0002
ECHS1	enoyl Coenzyme A hydratase, short chain, 1, mitochondrial	P30084	1892	**0.3**	0.0002
GATM	glycine amidinotransferase (L-arginine:glycine amidinotransferase)	P50440	2628	**0.3**	0.0003
ASS	argininosuccinate synthetase	P00966	445	**0.3**	0.0008
ATP5H	ATP synthase, H+ transporting, mitochondrial F0 complex, subunit d	O75947	10476	**0.4**	0.0010
HSPD1	heat shock 60kDa protein 1 (chaperonin)	P10809	3329	**0.4**	0.0019
ATP5D	ATP synthase, H+ transporting, mitochondrial F1 complex, delta subunit	P30049	513	**0.3**	0.0029
PCK2	phosphoenolpyruvate carboxykinase 2 (mitochondrial)	Q16822	5106	**0.3**	0.0033
CALB1	calbindin 1, 28kDa	P05937	793	**0.3**	0.0035
BHMT	betaine-homocysteine methyltransferase	Q93088	635	**0.5**	0.0041
ACY1	aminoacylase 1	Q03154	95	**0.4**	0.0043
AKR1A1	aldo-keto reductase family 1, member A1 (aldehyde reductase 2ALR)	P14550	10327	**0.4**	0.0054
AGMAT	agmatine ureohydrolase (agmatinase)	Q9BSE5	79814	**0.5**	0.0064
ACAT1	acetyl-Coenzyme A acetyltransferase 1 (acetoacetyl Coenzyme A thiolase)	P24752	38	**0.4**	0.0078
GSTO2	glutathione-S-transferase, alpha-class, omega 2 subunit	P08263	119391	**0.4**	0.0101
PKM2	pyruvate kinase, muscle	P14618	5315	**14.7**	0.0170
ANXA4	annexin A4	Q6P452	307	**2.7**	0.0225
ANXA5	Annexin V	P08758	308	**2.3**	0.0245
HSPB1	heat shock 27kDa protein 1	P04792	3315	**2.4**	0.0256
FABP7	fatty acid binding protein 7, brain	O15540	2173	**6.3**	0.0262
TBB2	tubulin, beta polypeptide	P07437	7280	**19.5**	0.0264
ENO2	enolase 2, (gamma, neuronal)	P09104	2026	**32.8**	0.0364
LDHA	lactate dehydrogenase A	P00338	3939	**3.8**	0.0383
CLIC1	chloride intracellular channel 1	O00299	1192	**2.8**	0.0414
GAPDH	glyceraldehyde-3-phosphate dehydrogenase	P04406	2597	**2.1**	0.0450
PSME2	proteasome (prosome, macropain) activator subunit 2 (PA28 beta)	Q9UL46	5721	**2.2**	0.0460
CSTB	cystatin B (stefin B)	P04080	1476	**4.0**	0.0464
HINT1	histidine triad nucleotide binding protein 1 (PKCi substrate analog)	P49773	3094	**2.4**	0.0482

### Confirmation of proteomic analysis: identification of Hsp27 and PKM2

**Figure 3:**
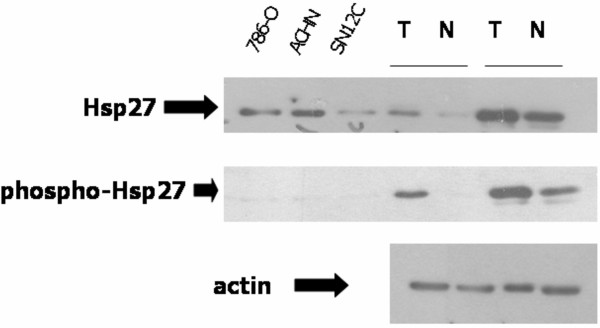
Anti-apoptotic proteins are upregulated in ccRCC as confirmed by immunoblotting. Three RCC cell lines, and 2 tumors which were used in the proteomic analysis were immunoblotted with Hsp27 or phospho-Hsp27 antibodies; actin is a loading control. Solid line indicates same kidney.

**Figure 4:**
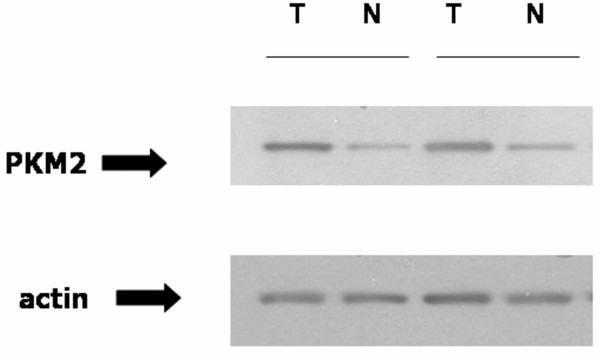
The HIF-1 target, PKM2, is increased in ccRCC as confirmed by immunoblotting. Two tumors which were used in the proteomic analysis were immunoblotted with PKM2 antibody; actin is a loading control. Solid line indicates same kidney.

To confirm that the proteomic analysis utilized was indeed valid, we performed further analysis of two of the highly upregulated spots that were identified by MS as Hsp27 and PKM2 (Figs. 3, 4). The Hsp27 protein, which lies downstream of p38MAPK, is a member of the heat-shock class of proteins which play pivotal roles in a variety of cellular processes such as stress and apoptosis. Hsp27 is of particular interest to our laboratory because it has been described to have anti-apoptotic functions[8] and lies downstream of p53 [9], similar to what we and others have described for p21. [10-12].

Hsp27 abundance was increased when assessed by immunoblotting (Fig. 3) and immunohistochemistry (Supplemental Fig. 2) of these tumors, confirming the proteomics data. In addition, two representative RCC tumors and adjacent normal tissue, and three RCC cell lines (786-0, ACHN and SN12C) were examined for Hsp27 and phospho-Hsp27. While none of the kidney cancer cell lines showed phospho-Hsp27, both of the tumors showed a high degree of phosphorylation of Hsp27 as compared to the adjacent normal tissue (Fig. 3a). This result is consistent with a lower than predicted isoelectric point of Hsp27 on 2D gels, which already indicated that the up-regulated Hsp27 is phosphorylated (theoretical pI = 6.0; actual pI on 2D gels = 5.3). Because the phospho-peptide does not ionize well, we were not able to observe it directly in the mass spectra.

We next examined changes in the levels of PKM2 in ccRCC and control tissues by immunoblotting (Fig. 4). In the absence of pVHL, as in VHL-deficient RCCs occurring in VHL disease, HIF-1α is constitutively activated (due to lack of degradation), such that these tumors behave as though they are constitutively hypoxic even though they are in fact flush with oxygen[1]. PKM2 is of special importance in RCC, since it is transcriptionally activated by HIF-1. Furthermore, hypoxic treatment of various cancer cell lines result in increased PKM2 mRNA[13], suggesting that this protein may be important in the HIF-1 response, as is most pronounced in VHL-deficient RCCs. Confirming our proteomic analysis, we found that PKM2 is markedly increased in the tumor tissues examined (Fig. 4).

Many of the other proteins we have identified by 2-dimensional gel analysis as altered in ccRCC have been confirmed in other published studies in RCC as well as other cancers, attesting to the validity of our analyses; for this reason, we have chosen not to confirm any additional protein changes by single protein immunoblotting.

### Network, pathway, and process analyses of significantly changed proteins in RCC

**Figure 5:**
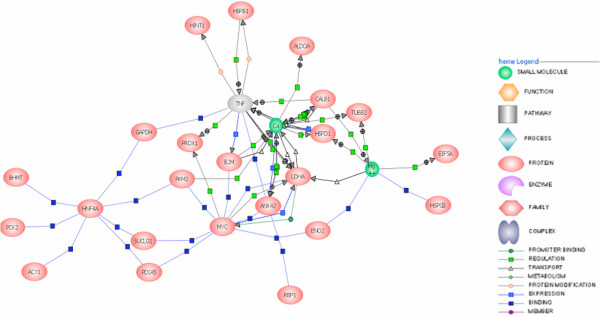
Network of interactions between the 31 differentially expressed proteins. TNF is not one of the 31 proteins but is shown here as it interacts with many components of the network. Nodes represent proteins and links indicate known interactions or modulations from neighboring nodes. Proteins at the bottom of the figure are in the 31 protein set but are not involved in the network. Links scheme -gray square: regulation; -green square: direct regulation; -green circle: promoter binding; -green open square: molecular transport; -blue open square: molecular synthesis; -blue square: expression; -yellow circle: protein modification; -purple circle: binding.

The 31 proteins which we identified by mass spectrometry with p value < 0.05 are listed in Table 2 with their associated molecular function and biological process(es). Most of these proteins have previously been described as involved in one or several cancer types (Table 2). They also have known interactions amongst themselves and most form a biological network as illustrated by the software Pathway Architect (Stratagene) (Fig. 5). Interestingly, network analysis pointed to the involvement of TNFα in ccRCC pathogenesis. Such association has been previously reported[14,15], and in this manner, our network analysis can reveal signaling molecules that are likely to be involved in the disease process but which are not identified in our analytical assays. This analysis, in particular, suggests further examination of the use of clinically available TNFα inhibitors (such as thalidomide and etanercept) for treatment of ccRCC.

**Table 2:** Annotation of proteins which were significantly altered in ccRCC as compared to norml tissue.

**Symbol**	**Gene**	**Entrez Gene ID**	**Biological Process**	**Molecular Function**	**Associated Cancer**
ACY1	aminoacylase 1	95	Amino acid biosynthesis	Hydrolase;Metalloprotease	
ASS	argininosuccinate synthetase	445	Amino acid biosynthesis;Nitrogen metabolism	Ligase	liver cancer
AGMAT	agmatine ureohydrolase (agmatinase)	79814	Amino acid catabolism	Hydrolase	
BHMT	betaine-homocysteine methyltransferase	635	Amino acid metabolism	Methyltransferase	
GATM	glycine amidinotransferase (L-arginine:glycine amidinotransferase)	2628	Amino acid metabolism	Transferase	

CLIC1	chloride intracellular channel 1	1192	Anion transport;Other homeostasis activities	Anion channel	breast and ovarian cancers
PRDX1	peroxiredoxin 1	5052	Antioxidation and free radical removal	Peroxidase	breast, liver and pancreatic cancers
GSTO2	glutathione-S-transferase, alpha-class, omega 2 subunit	119391	Detoxification;Antioxidation and free radical removal	Transferase	ovarian cancer

ECHS1	enoyl Coenzyme A hydratase, short chain, 1, mitochondrial	1892	Carbohydrate metabolism;Fatty acid beta-oxidation;Coenzyme metabolism;Vitamin biosynthesis	Synthetase;Dehydrogenase;Acetyltransferase;Acyltransferase;Hydratase;Epimerase/racemase;Ligase	liver cancer
PCK2	phosphoenolpyruvate carboxykinase 2 (mitochondrial)	5106	Gluconeogenesis	Decarboxylase	liver cancer
AKR1A1	aldo-keto reductase family 1, member A1 (aldehyde reductase 2ALR)	10327	Glucose metabolism; aldehyde metabolism	Reductase	breast cancer
ALDOB	aldolase B, fructose-bisphosphate	229	Glycolysis	Aldolase	liver cancer
ENO2	enolase 2, (gamma, neuronal)	2026	Glycolysis	Lyase	breast, lung and prostate cancers
GAPDH	glyceraldehyde-3-phosphate dehydrogenase	2597	Glycolysis	Oxidoreductase	liver, lung and ovarian cancers
LDHA	lactate dehydrogenase A	3939	Glycolysis	Dehydrogenase	breast and ovarian cancers
PKM2	pyruvate kinase, muscle	5315	Glycolysis	Carbohydrate kinase	ovarian cancer
SUCLG1	succinate-CoA ligase, GDP-forming, alpha subunit	8802	Tricarboxylic acid pathway	Synthetase	breast cancer

FABP7	fatty acid binding protein 7, brain	2173	Lipid and fatty acid transport;Lipid and fatty acid binding; Vitamin/cofactor transport;Steroid hormone-mediated signaling;Transport;Ectoderm development	Transfer/carrier protein	breast cancer
ANXA4	annexin A4	307	Lipid, fatty acid and steroid metabolism	Transfer/carrier protein;Annexin	pancreatic cancer
ANXA5	Annexin V	308	Lipid, fatty acid and steroid metabolism	Transfer/carrier protein;Annexin	breast and pancreatic cancers

ACAT1	acetyl-Coenzyme A acetyltransferase 1 (acetoacetyl Coenzyme A thiolase)	38	Protein acetylation	Acetyltransferase	liver cancer
HSPD1	heat shock 60kDa protein 1 (chaperonin)	3329	Protein folding;Protein complex assembly	Chaperonin	lung cancer
HSPB1	heat shock 27kDa protein 1	3315	Protein folding;Stress response	Chaperones	Colon, breast, ovarian cancer and pancreatic cancers
HSPCB	heat shock 90kDa protein 1, beta	3326	Protein folding;Stress response	Hsp 90 family chaperone	
CSTB	cystatin B (stefin B)	1476	Proteolysis	Cysteine protease inhibitor	prostate cancer
PSME2	proteasome (prosome, macropain) activator subunit 2 (PA28 beta)	5721	Proteolysis	Miscellaneous function protein	breast cancer

CALB1	calbindin 1, 28kDa	793	Calcium mediated signaling;Calcium ion homeostasis	Select calcium binding protein	
TBB2	tubulin, beta polypeptide	7280	Intracellular protein traffic;Chromosome segregation;Cell structure;Cell motility	Protein polymerization	breast cancer

ATP5D	ATP synthase, H+ transporting, mitochondrial F1 complex, delta subunit	513	Nucleoside, nucleotide and nucleic acid metabolism;Electron transport;Cation transport	Hydrogen transporter;Synthase;Other hydrolase	lung cancer
ATP5H	ATP synthase, H+ transporting, mitochondrial F0 complex, subunit d	10476	ATP synthesis coupled proton transport	ATP synthase	lung and ovarian cancer
HINT1	histidine triad nucleotide binding protein 1 (PKCi substrate analog)	3094	Signal transduction	Nucleotide phosphatase	breast cancer

**Figure 6:**
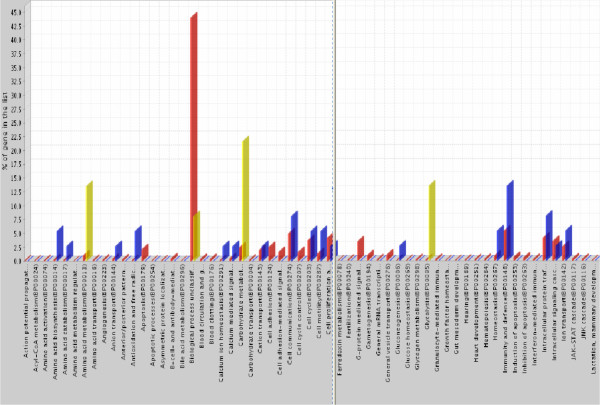
Significant Panther Biological Process (p < 0.01) shown in yellow. The RCC 31 protein set is compared to a human reference set of 23,401 translated transcripts and allocated in 242 biological process classes. Two sections of the histogram, encompassing glycolysis and amino acid metabolism pathways, are shown here; the complete histogram is available in Supplemental Fig. 3. Red bars correspond to percentage of proteins attributed to a given process, when looking at the 23,401 human reference set. Blue bars indicate the percentage of proteins from the RCC 31 protein sets in that class. Yellow bars indicate the process which we identified from our proteomic analysis to have a significance greater than 0.01. Glycolysis is the most significant pathway identified by this analysis.

We next used statistical tools to analyze the biological processes and molecular functions as well as the pathways which encompass the 31 significantly differential proteins in Table 1. Using the Panther HMM algorithm based on homology and trained on known proteins, we identified key processes associated with our 31 protein series (Table 3). After adjusting the p-value with Bonferroni correction for multiple testing, we found that glycolysis (4 E-05), carbohydrate metabolism (3 E-04) and amino acid metabolism (5 E-04) are the only processes with significant p-values (< 1 E-02) among the 242 Panther biological processes (Fig. 6 and Supplemental Fig. 3). Similar analysis indicates lyase as the only prevalent Panther molecular function (2 E-03), with the proteins aldolase ALDOB, lyase ENO2, decarboxylase PCK2 and hydratase ECHS1.

**Table 3:** Enriched processes identified with the Panther libraries using proteins which were significantly altered in ccRCC as compared to normal tissue, with p < 0.05. The pvalue is then adjusted with Bonferroni correction for multiple testing.

**Biological Process (Panther)**	**RCC Proteins**	***H. sapiens *Ref. **	**pValue**
Glycolysis	4	41	4.20E-05
Carbohydrate metabolism	7	584	2.90E-04
Amino acid metabolism	5	238	4.60E-04

A different approach using statistical tools on the Jubilant Pathart database yielded similar results (Table 4), with the most significant pathways also including carbohydrate and amino acid metabolism. As in the Panther analysis, glycolysis is again the most significant with p value < 1 E-05. The Jubilant database contains a greater number of metabolic processes than the Panther database, and several other carbohydrate metabolic pathways, such as propanoate, pyruvate, butanoate metabolism, urea cycle and citrate cycle, are identified with a significant p value < 5 E-03. In addition to arginine and proline metabolism, lysine degradation, valine, leucine and isoleucine degradation are also identified. The only significant non-metabolic pathway is the p53-mediated pathway with 6 proteins among the 31 proteins, yielding a p value < 4 E-04 (*vide infra*). The six proteins, lactate dehydrogenase (LDHA), glyceraldehyde 3-P dehydrogenase (GAPDH), Hsp27 (HSPB1), proteasome activator subunit 2 (PSME2), pyruvate kinase (PKM2) and the annexins A4 and A5 (ANXA4 and ANXA5) have all been associated to at least one type of cancer (breast, colon, kidney, liver, lung, ovarian, pancreatic); this association is further confirmatory regarding the veracity of our data and analyses.

**Table 4:** Enriched processes and pathways identified with the Jubilant PathArt database using proteins which were significantly altered in ccRCC as compared to normal tissue, with p< 0.05.

**Process or Pathway (Jubilant PathArt)**	**RCC Proteins**	***H. sapiens *Ref. **	**pValue**
Glycolysis/Gluconeogenesis	7	34	< 1.00E-05
Propanoate Metabolism	5	33	1.00E-05
Pyruvate Metabolism	5	37	1.00E-05
Arginine And Proline Metabolism	4	62	2.00E-05
Urea Cycle	4	38	2.00E-05
p53 Mediated Pathway	6	154	3.20E-04
Butanoate Metabolism	3	41	0.00196
Citrate Cycle	3	43	0.00224
Sterol, Vitamin K, Vitamin E, Carotenoids Biosynthesis	3	46	0.00271
Lysine Degradation	3	54	0.00422
Valine, Leucine And Isoleucine Degradation	3	56	0.00466
Purine Metabolism	4	121	0.00582
FAS Signaling Pathway	3	136	0.04344

**Figure 7:**
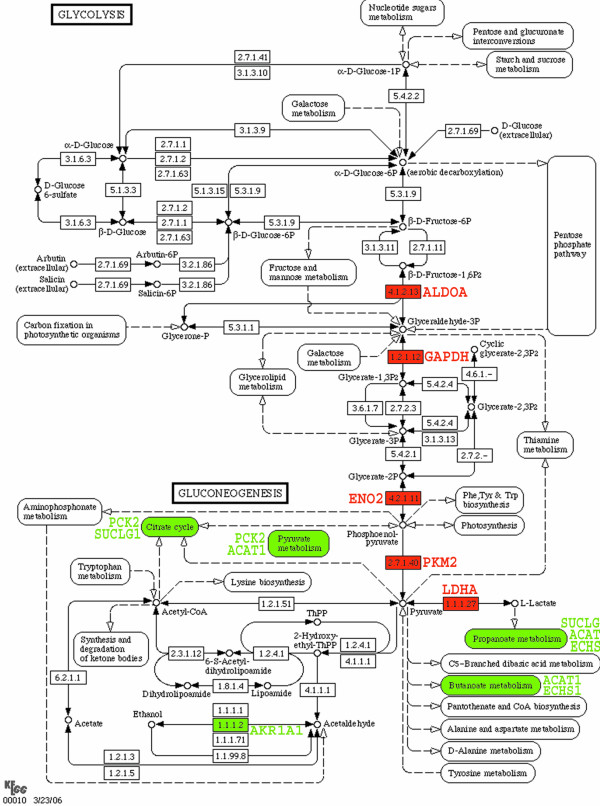
Proteins differentially regulated in RCC involved in carbohydrate metabolism are shown overlaid on the glycolysis and gluconeogenesis pathway KEGG #00010 diagram. Anaerobic glycolysis is upregulated while other carbohydrates metabolism appears down regulated. The enzymes colored in red correspond to the proteins which we found upregulated in ccRCC, and in green those (or pathways associated) we found downregulated in ccRCC. We added the corresponding gene symbol next to the enzyme or enriched process.

We next sought to integrate our data into a known extant pathway scheme, and for this analysis we chose the most significantly enriched pathway which we identified in this study. As shown in the KEGG glycolysis and gluconeogenesis diagram (Fig. 7), those glycolysis enzymes which we identified among the 31 altered proteins are all upregulated, including ALDOA (aldolase A) with p value = 0.051 (Supplemental Table 1) but excluding ALDOB (aldolase B), a fructose-bisphosphate which we found was downregulated with p value < 0.01. Lactate dehydrogenase, which activity in general is linked to hypoxia, is upregulated. In contrast, several carbohydrate metabolic pathways closely associated with glycolysis, such as pentanoate metabolism, pyruvate metabolism, and citrate cycle, are all down regulated. This is true also for the other significantly enriched pathways, arginine and proline metabolism and the urea cycle, both with enzymes (ASS, GATM, AGMAT and ACY1) being down regulated (Fig. 7).

The only significantly enriched non-metabolic pathway is the p53 mediated pathways (p value = 4 E-05) with 6 proteins among our 31 identified proteins (HSPB1, PKM2, PSME2, ANXA4, ANXA5, GAPDH and LDHA). This is not unexpected given the pivotal role played by the tumor suppressor proteins in DNA damage repair and, consequently, response to chemotherapy (reviewed in [10]). Elements of the pathway have been targeted by our laboratory and others as possible chemotherapy sensitizers in other cancers[10,12], and may be relevant to RCC as well. Thus this finding is not only confirmatory about the veracity of our data, but supports the continued investigation of mTOR inhibitors for ccRCC; these drugs act through attenuation of an element of the p53 pathway, p21[16], which is anti-apoptotic[11], pro-proliferative[17], and has prognostic value in ccRCC[5].

### Transcriptomic validation of proteomics results

There are several existing published studies on transcriptomic analysis of RCC; while not repeating such genomic studies, we used the microarray data generated by Takahashi *et al*[18] to confirm our results. We re-annotated the genes, which these investigators had determined to be significantly differential when comparing mRNA expression from RCC and normal renal tissue, using the most updated annotation database available. From these results, we generated a list of 88 genes with the NCBI Entrez identifier and used this list for pathway analysis with the Jubilant PathArt database. While only seven genes from this list correspond to the proteins identified in our study (ENO2, ALDOB, CALB1, ASS, ACY1, SUCLG1, GATM), the process analysis yielded remarkably similar results to our proteomic results (Table 5), with carbohydrate metabolism and amino acid metabolism being the most significant pathways, in particular glycolysis, and arginine and proline metabolism (p = 1 E-05). Urea cycle and citrate cycle were also significant in this analysis, as well as those for sterol, vitamin K, vitamin E and carotenoid biosynthesis. This concordance of transcriptomic data with our proteomic data is further validation of its veracity.

**Table 5:** Enriched pathways identified with Jubilant PathArt database, using genes identified in ccRCC microarray experiment published by Takahashi *et al *[18].

**Pathways (Jubilant PathArt)**	**RCC Genes**	***H. sapiens *Ref.**	**pValue**
Glycolysis/Gluconeogenesis	8	49	< 1.00E-05
Arginine And Proline Metabolism	7	92	1.00E-05
Urea Cycle	5	48	2.00E-05
Phenylalanine Metabolism	3	18	1.80E-04
Lysine Degradation	4	54	3.20E-04
Valine, Leucine And Isoleucine Degradation	4	56	3.70E-04
Nicotinate And Nicotinamide Metabolism	3	32	9.70E-04
Propanoate Metabolism	3	33	0.00106
Starch And Sucrose Metabolism	3	40	0.00183
Citrate Cycle	3	43	0.00224
Sterol, Vitamin K, Vitamin E, Carotenoids Biosynthesis	3	46	0.00271
Terpenoid Biosynthesis	2	13	0.00280
Phenylalanine, Tyrosine And Tryptophan Biosynthesis	2	17	0.00476
Purine Metabolism	4	121	0.00582
Cysteine Metabolism	2	21	0.00718
Pantothenate And CoA Biosynthesis	2	21	0.00718
Tyrosine Metabolism	3	67	0.00754

### Urinary metabolic profiling verifies an identified altered pathway

Because some of the processes identified above may result in metabolic ''signatures'' in the urine which would be useful for RCC diagnosis as well as therapeutic responsiveness, we next performed a pilot study by metabolic profiling of several urines from RCC patients in an attempt to identify metabolites which are expected to result from activation of the enzymes involved in the above processes. We focused on intermediate or end-products of the glycolysis pathways, since this is expected based on the process analysis described above.

We identified 40 primary metabolites in the urine of 5 ccRCC and 5 control patients (distinct from those patients from whom the kidney tissue was obtained). While no phosphorylated intermediates were present in urine, we were able to identify a variety of small molecule glycolytic intermediates, such as glucose, pyruvate, sorbitol, and succinate, and TCA cycle intermediates such as malate and aconitate but not oxaloacetic acid, fumarate, citrate and isocitrate. From these 40 metabolites, only the sorbitol level was significantly altered at p = 0.02 with a 5.4-fold higher level in the ccRCC patients as compared to control samples (Table 6).

**Table 6:** Primary metabolites differentially expressed in urine of ccRCC and normal control patients (creatinine not shown).

**Compound**	**pValue**	**Mean Fold Change**
**Sorbitol**	**0.02**	**5.4**

Glycerol	0.08	3.7
Palmitate	0.08	2.8
myo-Inositol	0.1	11.5
Pelargonic acid	0.1	2.4
Gluconic acid	0.16	1.9
Fructose	0.16	1.7
Stearic acid	0.18	1.7
Hippuric acid	0.22	0.5
Mannitol	0.24	1.4
N-Acetylglucosamine	0.26	1.6
Malic acid	0.3	5.7
Ethanolamine	0.32	0.7
Galactose	0.33	1.6
Threonic acid	0.34	1.5
Sucrose	0.35	0.7
1,6-anhydro-beta-glucopyranose	0.36	0.6
m-Cresol	0.37	0.6
Quinic acid	0.37	0.6
Ribose	0.39	1.5
Capric acid	0.47	2.1
Erythronic acid	0.51	1.6
Xylose	0.54	1.3
Glucose	0.54	0.8
5-ε-Hydroxybutylhydantoin	0.55	1.4
Xylitol	0.56	1.5
Threitol	0.59	1.2
Ribitol	0.6	1.3
Tetronate	0.63	1.2
4-Hydroxybenzoate	0.66	0.7
Succinate	0.69	0.7
4-Hydroxyphenylacetic acid	0.78	0.8
Oxoproline	0.82	0.9
Pyruvate	0.83	0.8
Gluconic acid	0.85	1.1
Rhamnose	0.86	0.9
Aconitic acid	0.87	0.9
Lactate	0.87	0.9
Erythritol	0.95	1

The use of creatinine as reference for urinary excretion volumes and metabolism is frequently questioned due to the biological variability of creatinine itself. When raw data are normalized to the sum of all detected metabolites instead solely to creatinine, mannitol and myo-inositol also become significantly increased in RCC patients (p < 0.05). Both compounds refer to sugar alcohol metabolism and indicate that a combined assay on reduced sugars may serve as stronger and more valid diagnostic biomarker than just a single compound alone. This finding is in accordance to the general anoxic state of cancer cells that favors reductive metabolism and thus may be indicated by reducing glucose directly to sugar alcohols in side reactions.

## Discussion

While a relatively infrequent malignancy, kidney cancer is distinguished by its being associated with notably unsatisfactory treatment options. Thus, the identification of biomarkers in easily accessible patient materials (such as blood and urine) is needed in order to identify affected patients while the disease is not metastatic and the tumor is still resectable. In this study, we have utilized several ''omic'' techniques to identify candidate pathways and networks which are altered in ccRCC and which can therefore be utilized in designing a diagnostic test for patients at higher risk for this disease, as well as to suggest novel therapeutic approaches. In light of the fact that reproducibility and variability of obtained data dictate optimal sample size in proteomics studies, our highly concordant results (validated by immunoblotting, high confidence p-values, and corroboration of our data with independently published proteomic and transcriptomic data) underscore the accuracy of our data, despite its relatively small sample size.

In order to confirm our proteomic analysis, we examined two separate proteins which were found to be significantly altered by 2D gel electrophoresis and MS identification. These two proteins were selected because they play key roles in oncogenesis and/or response to therapy as detailed below. Levels of Hsp27 have been reported to be elevated in kidney[19], breast[20], and liver[21] cancers, as has the phosphorylated form[22]. Using proteomic and then immunoblotting analysis in RCC, we have confirmed that phospho (but not native) Hsp27 is elevated in ccRCC in a parallel manner to p21. These findings were of special interest, since both proteins are induced by p53 [9], and there are reports that elevated levels of Hsp27[20], as p21[5], are associated with decreased patient survival. These data are also consistent with our pathway analysis showing that the p53 pathway is altered in ccRCC (Table 4).

Our proteomic analysis is not exhaustive, and is biased toward identification of high abundance soluble proteins as is normal for 2D gel-based approaches. Proteins with molecular masses higher than 150 kDa and lower than 15 kDa as well as proteins with isoelectric points outside the range of pH 3 – 10 are not identified. In addition, hydrophobic membrane proteins are underrepresented on 2D gels. Nevertheless, most cellular proteins have properties that make them amendable to the 2D gel approach, and liquid chromatography-based approaches (1D and 2D) have other pitfalls.Furthermore, high-abundance proteins which are altered in RCC are those which are most likely to have an impact on RCC-specific alteration of cellular phenotype. Finally, we performed comprehensive pathway analysis which allows us to identify the enriched biological networks, pathways, and processes involved, using only fractional information generated with the 2D gels, thereby alleviating at least some of the limitations of this technology.

Our pathway analysis has allowed us to identify groups of genes and proteins which are organized into metabolic and signaling pathways relevant to the oncogenesis or progression of ccRCC. The two different and independent methods used, Panther libraries and Jubilant PathArt, result in similar findings: glycolysis enzyme levels are the most significantly altered in ccRCC. This is in agreement with other studies in various cancers[23,24]. Also in agreement with these published results[23], we observed similar patterns of expression for the proteins aldolase-fructose-bisphosphate ALDOB and ALDOA, with the former being upregulated and the latter downregulated. Furthermore, while we have not performed a *de novo *transcriptomic study on the same samples used for this proteomics analysis, we have examined and updated the microarray data on ccRCC obtained by Takahashi *et al*[18] and found that these data are consistent with our proteomic results. All of these concordant results underscore the pertinence of our data, despite the fact that it has been generated from a relatively small sample set.

In this study, we show with a high degree of statistical confidence that other pathways closely associated with gluconeogenesis, such as pyruvate metabolism, pentanoate metabolism, butanoate metabolism, as well as arginine and proline metabolism and the urea cycle, are downregulated in ccRCC. In contrast, as for pyruvate being a substrate, we observed an increase in lactate dehydrogenase (LDHA), which is known to be playing an active role in anaerobic glycolysis, thus reflecting the hypoxic conditions known to be present in proliferating cancer cells, especially RCC. LDHA increase has been shown in a variety of cancers but the hypothesis that LDHA is involved in an apoptotic pathway[25] could imply a more complex role of this enzyme in ccRCC.

We have correlated our transcriptomic and proteomic results with an analysis of metabolites in the urine and found compounds which could result from activation of the carbohydrate metabolism pathways, in particular the glycolysis and gluconeogenesis pathways; such urine metabolites could conceivably be utilized as part of a screening procedure for RCC, as we describe. Metabolomic analysis in principle has considerable promise for translation of basic science data to the clinic in a variety of diseases[26]. Nephrologic disorders are particularly amenable to metabolomic analysis, since the urine is the final repository for a number of metabolites. However, since metabolomic analysis is quite dependent on a number of variables such as diet and medications, detection of the pathways involved in this pathology, and which theoretically result in identifiable metabolites, increases the chance of success in this type of analysis. Our finding that, of the 40 metabolites profiled in the urine, sorbitol was significantly elevated in the ccRCC patients' urine suggests that the sorbitol pathway of glucose metabolism is active in the RCC kidneys. While sorbitol acts as an intracellular osmolyte to protect medullary cells from the hypertonic extracellular milieu (see below)[27], activation of the sorbitol pathway is also seen in states of hyperglycemia, and thus in states in which glycolysis is active[28]. This is consistent with our finding of elevated glycolysis pathway enzymes by our proteomic (and historical genomic) analysis; however, this data awaits confirmation in a larger sample size.

Sorbitol is one of the small organic solutes (osmolytes) that are accumulated within the cells of the renal medulla and protects these cells against high medullary tonicity. Thus, it is possible that sorbitol is altered due to a change in osmolality of the urine[27]. However we measured urine osmolality in RCC and control urines (data not shown) and did not find a significant difference, arguing against (but not disproving) this mechanism. Sorbitol may be increased as a result of non-specific derangement of kidney cell osmolar function. However, it is also possible that sorbitol is being produced by alternate glycolysis pathways in the tumors and that our observation of decreased aldehyde reductase activity in the RCCs reflects feedback inhibition of expression of this enzyme. Such enzymes are part of the aldo-keto reductase super family and represent monomeric NADPH-dependant oxidoreductases that have a wide substrate specificity for carbonyl compounds[29]. This is of some interest, as it has been shown that sorbitol causes resistance to some chemotherapeutic agents[30], such that its production by the RCC tumors that we examined in this study may be a mechanism of chemoresistance. Whether there are other pathophysiological functions for sorbitol or its pathway enzymes in RCC is unknown but currently under active investigation in our laboratory.

It has indeed been known since the 1920's that advanced tumors have high rates of glycolysis[31], however, translating this finding into a diagnostic assay has not, to our knowledge, been attempted. Using two independent approaches, we demonstrate in this study that glycolysis related enzymes played a major role in the metabolism of RCC, and our findings that there appears to be a metabolic "signature" in the urine of activation of this pathway is the first such report. It is possible, of course, that this urinary signature is not unique to RCC but may be the result of the presence of any malignancy, given the known high glycolysis rates[31,32]. In addition, this may be an effect intrinsic to the kidney, although this is unlikely given the significant difference between malignant and control tissue. These studies are currently underway in our laboratories.

In this study, we utilized proteomic analysis of tumors to determine which pathways and processes are likely to be operative in kidney cancer, and, supporting our findings, extant genomic analysis from other laboratories is consistent with our data identifying the glycolysis pathway as being significantly altered in ccRCC. We utilized these identified pathways to discover a metabolic signature in the urine of ccRCC patients as products of glycolysis and sugar alcohol metabolism. Thus, in this study, we have taken a systems approach to RCC, utilizing proteomics to identify pathways altered in this disease, confirming our results with existing transcriptomic data, and then successfully identifying a metabolic signature in the urine of RCC patients. While levels of single small metabolites may lack diagnostic specificity, subsequent studies of more patients and additional metabolites may lead to patterns of metabolites whose appearance will lead to novel urinary diagnostic tests for ccRCC in high risk patients. In addition, alterations to these pathways (especially the p53 and FAS signaling pathways, see Table 4) will allow clinicians to better tailor therapies (such as DNA-damaging chemotherapies and mTOR inhibition as discussed above) to specific patients, as well as to monitor the molecular effects of therapy prior to gross tumor changes.

## Conclusion

In this study, we have used proteomic and metabolomic techniques to study tissue and urine, respectively, by network, pathway and process analysis in clear cell renal cell carcinoma patients to demonstrate those biochemical processes which are activated in the disease. Knowledge of these pathways will ultimately lead to novel assays for their metabolic signatures in patient biofluids, and we have begun to examine urine metabolomics to confirm this likelihood. Such assays will ultimately be useful for early diagnosis of disease in high risk patients as well as choice of, and response to, specific therapies.

## Methods

### Materials

Goat polyclonal Hsp-27 and rabbit polyclonal phospho-Hsp27 antibodies were obtained from Santa Cruz Biotechnology and used at a 1:1000 and 1:200 dilutions, respectively. Goat polyclonal PKM-2 antibody was obtained from Novus and used at a dilution of 1:1000. Horseradish peroxidase-conjugated anti-rabbit IgG and horseradish peroxidase-conjugated anti-mouse IgG was obtained from Bio-Rad and used at a 1:15000 dilution. Reagents for the Enhanced Chemiluminescence system were obtained from Amersham Pharmacia. All other reagents were from Sigma. RCC and adjacent control tissue was obtained from the UC Davis tissue bank after appropriate Institutional Review Board approvals (UCD IRB#200312072-3), and the urine samples from cancer patients were obtained from the Cooperative Human Tissue Network (CHTN) at Vanderbilt University.

### Western blots

The RCC cell lines 786-0, ACHN were obtained from ATCC, and SN12C as a kind gift from Dr. Isaiah J. Fidler. Equal protein quantities were electrophoresed and Western-blotted as described[33]. To confirm equal protein loading blots were either reprobed with β-actin or equal amounts of lysates were loaded in duplicate lanes in the same gel and separated after transfer to be probed for β-actin separately.

### Immunohistochemistry

Formalin fixed, paraffin embedded tissue blocks of the human kidney tumor samples were sectioned at 4-5 micron thickness, mounted on charged glass slides and baked for one hour at 60°C. Slides were deparaffinized with 3 changes of xylene, and the endogenous peroxidases were quenched with hydrogen peroxide, followed by a series of ethanol rinses (100%, 100%, 95%, and 70%). Slides were rehydrated and prepared for antigen retrieval with citrate buffer and blocked with 10% goat serum diluted in PBS. After incubation with phospho-Hsp27 antibody (1:50) in PBS + 0.05% BSA overnight, slides were rinsed in PBS and incubated with anti-goat secondary antibody (Jackson ImmunoResearch 1:1000), and incubated with DAB (Vector) following vender instructions. Slides were counterstained in Mayer's hematoxylin, dehydrated, cleared, and coverslipped. Slides were photographed with a Zeiss Axioskop light microscope and Axiocam digital camera

### Two-dimensional gel electrophoresis and spot analysis

Proteins were extracted from frozen tissue as previously described[34]. A total protein concentration of 600 μg in IPG Rehydration Buffer containing 15 mM DTE and 0.5% ampholytes pH 3–10 (Amersham Biosciences) was loaded on 17-cm IPG strips pH 3–10 non-linear (NL) from Bio-Rad (Hercules, CA) using passive rehydration at 20°C. Isoelectric focusing was performed using the Protean IEF cell (Bio-Rad) for 65,000 Vh. After equilibration IPG strips were loaded on uniform 11% polyacrylamide/bis-acrylamide gels (Protean Xi, Bio-Rad) and electrophoresed at 20 mA constant current at 10°C. Gels were stained with Colloidal Coomassie blue and scanned with an Epson 1680 Scanner as described previously[7]. Spot quantification and statistical analysis of differences in spot values were done as previously described[34]. The protein spots were matched between gels using the All-to-One warping strategy using the Delta 2D gel analysis software from Decodon GmbH (Greifswald, Germany). One RCC sample gel was selected as the reference gel and all replicates of all conditions were matched to this gel using the exact warping method between each gel pair with defined vectors from sample to Master gel. In order to ensure that the same spot area was quantified in all gels, a master gel was created by fusing all gel images with the maximum intensity option selected in Delta2D. Subsequently, the spots in the master gel were detected, using optimized spot detection parameters with exact spot outlines. In some cases spot outlines were manually edited to separate spots or to eliminate background interference. The detected spots from the Master gel were then transferred to all other gels, instead of individually quantifying each gel, which yielded different spot outlines. To further ensure uniformity between replicates and to minimize gel-to-gel variation due to experimental conditions, the volume of each detected spot was normalized to the sum total of the volumes of ten internal standard spots (std 110), selected as spots present at visually uniform intensity in all gels and whose total sum ranged between 2 and 4% of the total spot volume in each gel. The standard deviation of each quadruplicate determination was calculated based on the absolute spot volumes normalized to the sum of the internal standards. All further statistical analyses were performed with Excel using paired RCC and normal sample spot volume values, normalized to the sum of internal standards as above. To determine if an equal or unequal variance existed between variances of RCC and normal sample spot volumes, an F-test was performed with Alpha:0.05. If the resulting P(F f) was less than 0.05, unequal variances were assumed; otherwise, equal variances between conditions were assumed. An ensuing paired t test with Alpha:0.05 was performed between spot volume means of RCC and normal samples on the basis of the results of the F-test. The corresponding P-value, P(T t), was reported as a measure of significant statistical variability between conditions.

Up- and down-regulated spots were extracted from gels and tryptic in-gel digestion and peptide extraction performed as previously described[34]. Each spot was placed in a single well of a ZipPlate™ (Millipore, Billerica, MA) containing immobilized C18 resin. Spot processing was performed at room temperature using reagents provided in the Montage In-Gel DigestZP Kit (Millipore) as previously detailed[34].

### MALDI-TOF/TOF mass spectrometry

MALDI-TOF/TOF analysis was performed as previously described[34]. Briefly, MALDI matrix α-cyano-4-hydroxycinnamic acid (HCCA, Sigma, St-Louis, MO) was recrystallized from 70:30 acetonitrile:H2O prior to use and eluted samples spotted in 0.5 μL-increments on a stainless steel MALDI plate. They were then overlaid with 2 × 0.5 μL of 2 mg/mL HCCA. Samples were analyzed on a 4700 Proteomics Analyzer from Applied Biosystems (Foster City, CA) using both MS and MS/MS operating modes. Peptide fragmentation in MS/MS mode was achieved either by post-source decay (PSD) or collision-induced dissociation (CID) using atmosphere as the collision gas. Protein identification was carried out with GPS Explorer software (Applied Biosystems) using the Mascot search algorithm and DeNovo Explorer modules included in the 4700 Explorer software (Applied Biosystems). The limit for mass accuracy was set at 50 ppm.

### Process and pathway analysis

We used two approaches based on the Panther libraries [35] and the Jubilant Biosys pathways analysis tool PathArt. The Panther libraries are based on multiple sequences alignments and Hidden Markov Models to classify uncharacterized proteins in protein families, functions and processes. Out of 23401 refseq genes of the human genome, 56% have been assigned to a Panther biological process and 57% to a Panther function. The Jubilant PathArt is a human curated database, containing pathways and diseases information based on published data in scientific journals. This dataset is updated quarterly and contains to date almost 50,000 interactions from over 2000 pathways. Both databases, Panther and PathArt, used simultaneously allows meaningful statistical evaluation of the process or pathway hits.

For the Panther analysis, the binomial statistics tool is used to compare classifications of multiple clusters of lists to a reference list to statistically determine over- or under- representation of Panther classification categories. Each list is compared to the reference list using the binomial test for each molecular function or biological process. The P-value is then adjusted with the Bonferroni correction for multiple testing as many statistical tests are performed at the same time. This correction multiplies the single-test P-value by the number of independent tests to obtain an expected error rate.

With Jubilant Biosys PathArt, a two-sided Fisher's exact test is used to calculate the P-value. A 2X2 contingency table is used and probabilities are calculated, where the rows of the table are the user inputs (number of genes in the pathway and number of genes not in the pathway), and the columns are data in the database (number of genes in the pathway and number of genes not in the pathway).

### Metabolic profiling

Due to insufficient enzyme selectivity, no urease treatment was performed prior to metabolite extraction (data not shown). Metabolite profiling of a select list of 39 identified compounds was carried out on a gas chromatography-time of flight mass spectrometer. 30 μl of urine was extracted with 0.5 mL of a solution of water/methanol/chloroform at 2:5:2 at -20°C. For GC-TOF MS (Leco Pegasus II GC-TOF mass spectrometer; Leco, St. Joseph, MI, USA) analysis, the organic phase was dried and dissolved in 20 μL of methoxamine hydrochloride (20 mg/mL pyridine) and incubated at 30°C for 90 min with continuous shaking. Then 180 μL of *N*-methyl-*N*-trimethylsilyltrifluoroacetamid (MSTFA) were added to exchange acidic protons at 37°C for 30 min. The derivatized samples were stored at room temperature for 120 min before injection. GC-TOF analysis was performed on an HP 5890 gas chromatograph with tapered, deactivated split/splitless liners containing glasswool (Agilent, Böblingen, Germany) and 1 μL splitless injection at 230°C injector temperature. The GC was operated at constant flow of 1 mL/min helium and a 40 m 0.25 mm id 0.25 μm RTX-5 column with 10 m integrated precolumn. The temperature gradient started at 80°C was held isocratic for 2 min, and subsequently ramped at 15°C/min to a final temperature of 330°C which was held for 6 min. Twenty spectra *per *second were recorded between *m/z *85–500. Peak identification and quantification were performed using the Pegasus software package (Leco) based on mass spectral comparison to an in-house library of authentic standards. Automated assignments of unique fragment ions for each individual metabolite were taken as default as quantifiers, and manually corrected where necessary. All artificial peaks caused by column bleeding or phtalates and polysiloxanes derived from MSTFA hydrolyzation were manually identified and removed from the results table. Metabolite peak areas were normalized to creatinine. Student's *t*-test was performed in Microsoft Excel 5.0.

## Competing interests

The author(s) declare that they have no competing interests.

## Authors' contributions

BP conceived of and performed the pathway and network analysis, and helped write and edit all versions of the manuscript. JL, NV, AD, and PYL carried out the analytical studies. OF performed metabolomic analysis of the urine. DK performed proteomic analysis and helped draft the manuscript. RHW conceived of the study and wrote the original and final versions of the manuscript.

## Supplementary Material

Additional file 1 supp data. Supplemental Fig. 1: Automatic annotation of de novo amino acid sequence on tryptic peptide m/z 1163 of HSP27. Supplemental Fig. 2: Immunohistochemical analysis of phospho-Hsp27. Supplemental Table 1: All identified proteins which are different in ccRCC compared to control tissue. Click here for file

Additional file 2 supfig3. The complete list of significant biological processes identified by Panther analysis. Click here for file
