# Supplementary material for: Pathway analysis of kidney cancer using proteomics and metabolic profiling
Source: Mol Cancer. 2006 Nov 24;5:64. doi: 10.1186/1476-4598-5-64 (PMC1665458; doi:10.1186/1476-4598-5-64)
Supplement: Additional file 2 — supfig3. The complete list of significant biological processes identified by Panther analysis. [file 1476-4598-5-64-S2.pdf]

PANTHER BAR CHART OF GENE COUNT (http://www.pantherdb.org/)

Display: Bar Chart of Gene Count   p-Value Cutoff (<=): 0.01   Highlight

PANTHER Classification System

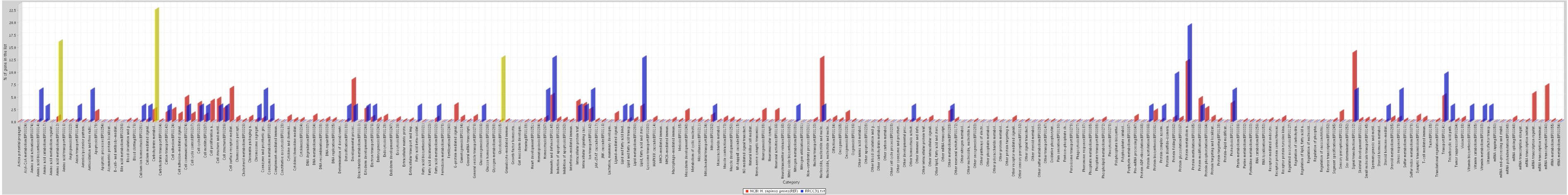

* Chart tooltips are read as: File name:Category name (Accession): # genes, Percent of gene hit against total # genes in the list, p-value
** % of gene list in the category is calculated for each testing list as: # genes for the category/ # total genes in the list * 100

© Copyright 2005 Applied Biosystems. All Rights Reserved.
