# Supplementary material for: Pathway analysis of kidney cancer using proteomics and metabolic profiling
Source: Mol Cancer. 2006 Nov 24;5:64. doi: 10.1186/1476-4598-5-64 (PMC1665458; doi:10.1186/1476-4598-5-64)
Supplement: Additional file 1 — supp data. Supplemental Fig. 1: Automatic annotation of de novo amino acid sequence on tryptic peptide m/z 1163 of HSP27. Supplemental Fig. 2: Immunohistochemical analysis of phospho-Hsp27. Supplemental Table 1: All identified proteins which are different in ccRCC compared to control tissue. [file 1476-4598-5-64-S1.pdf]

Supplemental Figure 1: Automatic annotation of *de novo* amino acid sequence on tryptic peptide m/z 1163 of HSP27 by ABI 4700 De Novo Explorer. The y ion series is shown in green, b ions in red, a ions in blue, and immonium ions and internal peptides in purple. The sequence determined from this spectrum LFDKAFGLPR correctly reflects the actual sequence LFDQAFGLPR of this peptide with the exception of amino acids that have very similar masses and cannot be distinguished by MALDI-TOF/TOF MS (K/Q).

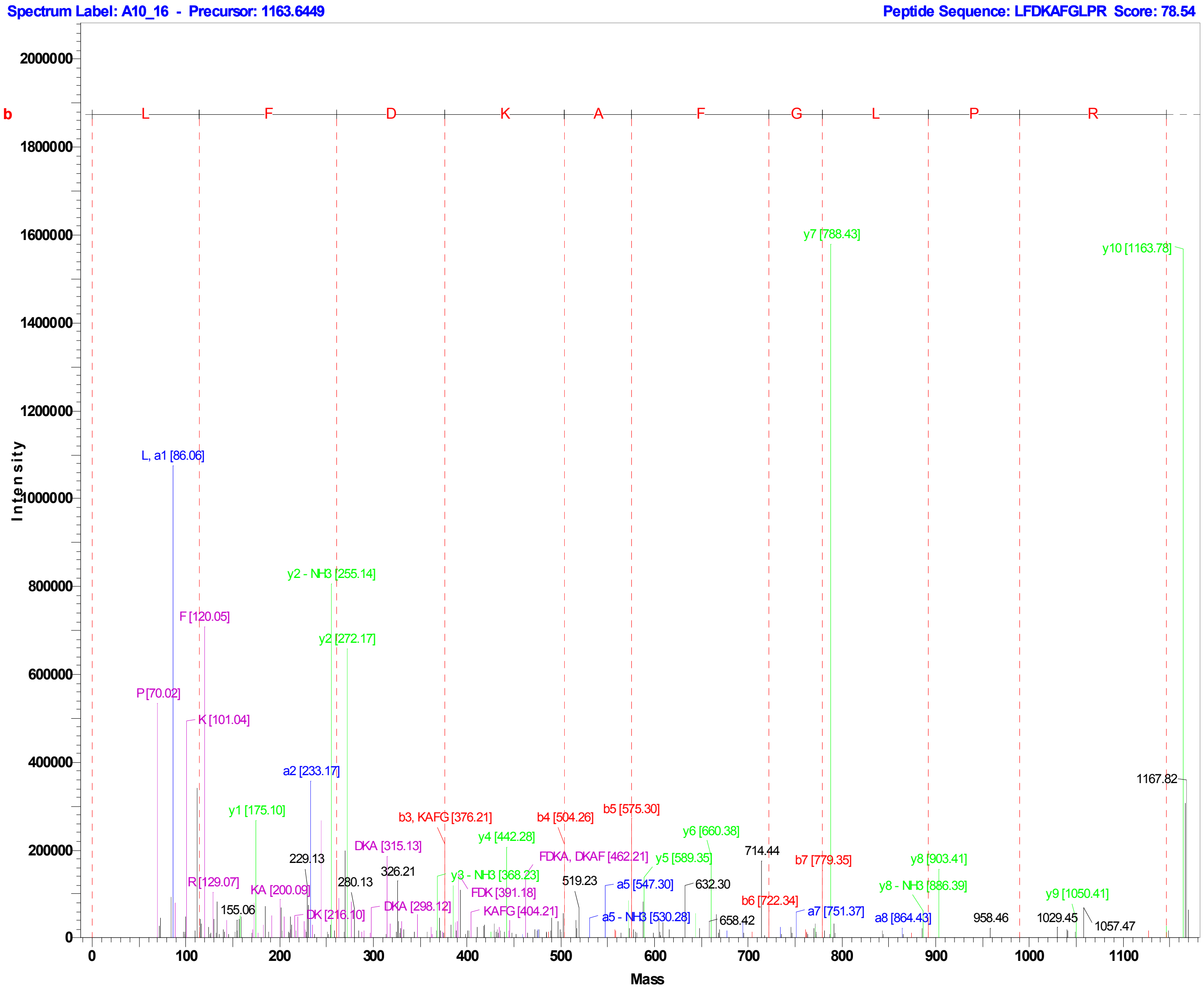

Supplemental Fig. 2: Immunohistochemical analysis shows increased nuclear staining of phospho-Hsp27 in ccRCC cells (left) as compared to normal tubular cells (right).

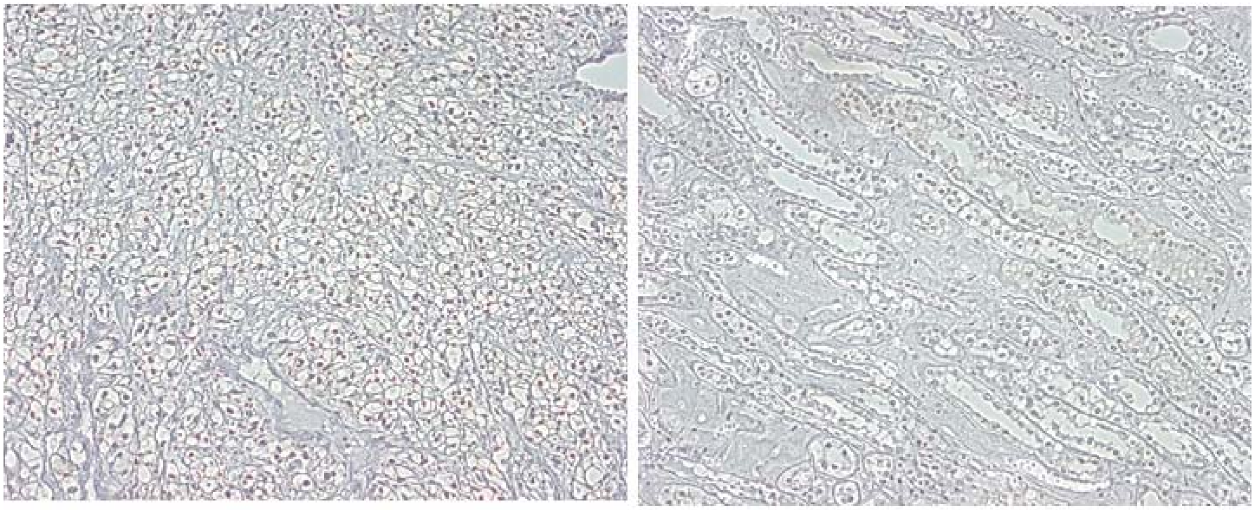

Supplemental Fig. 3: Enriched Panther biological processes (pvalue < 0.01). The RCC 31 protein set is compared to a human reference set of 23,401 translated transcripts and allocated in 242 biological process classes. Red bars corresponds to percentage of proteins attributed to a given process when looking at the 23,401 human reference set. Blue bars correspond to percentage of proteins from the RCC 31 protein sets. Yellow bars indicate the process which we identified from our proteomic analysis to have a significance greater than 0.01. Glycolysis is the most significant pathway seen by this analysis. (see separate file: SupFig3.pdf)

Supplemental Table 1: All identified proteins which were altered in ccRCC as compared to normal tissue

spot ID	Protein name	SwissProt ID	Entrez Gene ID	RCC:normal kidney	SEM	pValue	Mascot score	MS Seq. coverage
3up	enolase 2	P09104	2026	32.8	11.7	0.0364	442	32%
4up	beta-tubulin	P07437	7280	19.5	5.9	0.0264	425	36%
5up	coronin 1A (actin-binding protein)	P31146	11151	12.8	7.0	0.0960	469	42%
6up	pyruvate kinase 3	P14618	5315	14.7	3.7	0.0170	509	28%
8up	GA-3-PDH	P04406	2597	5.2	3.4	0.1523	325	34%
28up	Brain-type fatty acid binding protein	O15540	2173	6.3	1.7	0.0262	736	84%
2up	protein disulfide isomerase-related protein 5	Q15084	10954	2.2	0.5	0.0522	771	52%
7up	fructose-1,2, bisphosphate aldolase A	P04075	226	3.1	0.9	0.0515	731	37%
9up	GA-3-PDH	P04406	2597	2.1	0.5	0.0450	300	45%
10up	GA-3-PDH	P04406	2597	3.3	1.8	0.1488	303	49%
11up	L-LDH A	P00388	3939	3.8	1.0	0.0383	564	38%
12up	ANXA2 protein (Annexin A2)	P07355	302	2.2	0.6	0.0686	950	66%
13up	Annexin V	P08758	308	2.3	0.4	0.0245	424	54%
14up	chloride intracellular channel 1 variant (CLIC 1)	O00299	1192	2.8	0.7	0.0414	621	73%
16up	Proteasome activator complex subunit 2	Q9UL46	307	2.2	0.5	0.0460	447	57%
18up	Annexin A4	P09525	307	3.7	1.2	0.0540	881	68%
19up	Annexin A4	Q6P452	307	2.7	0.5	0.0225	165	28%
20up	heat shock protein 27	P04792	3315	2.4	0.4	0.0256	454	69%
26up	initiation factor 5A isoform 1	P63241	1984	2.3	0.6	0.0706	372	48%
56up	beta-microglobulin (BM)	P61769	567	3.1	1.0	0.0673	185	71%
57up	PKCi substrate analog	P49773	3094	2.4	0.5	0.0482	234	77%
58up	cystatin B	P04080	1476	4.0	1.0	0.0464	405	64%
1down	HSP60	P10809	3329	0.4	0.1	0.0019	748	39%
2down	HSP90 (fragment)	P08238	3326	0.4	0.0	0.0001	196	17%
5down	mitoch. phosphoenolpyruvate carboxykinase 2	Q16822	5106	0.3	0.1	0.0033	286	32%
7down	aminoacylase 1	Q03154	95	0.4	0.1	0.0043	450	40%
9down	aldehyde reductase	P14550	10327	0.4	0.1	0.0054	589	51%
11down	fructose-1,2, bisphosphate aldolase B	P05062	229	0.1	0.0	0.0001	473	49%
12down	fructose-1,2, bisphosphate aldolase B	P05062	229	0.3	0.1	0.0093	456	42%
13down	L-arginine:glycine	P50440	2628	0.3	0.0	0.0003	398	44%

	amidinotransferase							
14down	L-arginine:glycine amidinotransferase	P50440	2628	0.3	0.1	0.0013	600	42%
15down	L-arginine:glycine amidinotransferase	P50440	2628	0.2	0.1	0.0009	566	51%
16down	L-arginine:glycine amidinotransferase	P50440	2628	0.3	0.1	0.0021	443	37%
17down	L-arginine:glycine amidinotransferase	P50440	2628	0.5	0.1	0.0091	509	40%
18down	L-arginine:glycine amidinotransferase	P50440	2628	0.3	0.1	0.0005	370	28%
19down	betaine-homocysteine methyltransferase	Q93088	635	0.5	0.1	0.0041	559	28%
21down	arginine succinate synthetase	P00966	445	0.3	0.1	0.0008	475	54%
22down	acetyl-CoA acetyltransferase 1	P24752	38	0.4	0.1	0.0078	480	56%
27down	peroxiredoxin 1	Q06830	5052	0.3	0.0	0.0002	590	68%
29down	succinate CoA ligase, GDP forming, alpha-subunit	P53597	8802	0.2	0.0	0.0000	260	33%
33down	ATP synthase delta chain	P30049	513	0.3	0.1	0.0029	120	47%
35down	glutathione-S-transferase, alpha-class, omega 2 subunit	P08263	119391	0.4	0.1	0.0101	513	44%
40down	mitoch. H+ transporting ATP synthase, F0 complex, subunit d, isoform a	O75947	10476	0.4	0.1	0.0010	120	47%
41down	agmatine ureohydrolase (agmatinase)	Q9BSE5	79814	0.5	0.1	0.0064	367	50%
42down	mitoch. short-chain enoyl-CoA-hydratase 1	P30084	1892	0.3	0.0	0.0002	522	48%
43down	calbindin 1	P05937	793	0.3	0.1	0.0035	308	32%
